# Growth in achondroplasia including stature, weight, weight-for-height and head circumference from CLARITY: achondroplasia natural history study—a multi-center retrospective cohort study of achondroplasia in the US

**DOI:** 10.1186/s13023-021-02141-4

**Published:** 2021-12-23

**Authors:** Julie E. Hoover-Fong, Kerry J. Schulze, Adekemi Y. Alade, Michael B. Bober, Ethan Gough, S. Shahrukh Hashmi, Jacqueline T. Hecht, Janet M. Legare, Mary Ellen Little, Peggy Modaff, Richard M. Pauli, David F. Rodriguez-Buritica, Maria E. Serna, Cory Smid, Chengxin Liu, John McGready

**Affiliations:** 1 Greenberg Center for Skeletal Dysplasias, McKusick-Nathans Department of Genetic Medicine, Johns Hopkins University, 733 N. Broadway Suite 579, Baltimore, MD 21205 USA; 2 Present Address: Bloomberg School of Public Health, Johns Hopkins University, Baltimore, MD USA; 3 Nemours/AI duPont Hospital for Children, Wilmington, DE USA; 4 McGovern Medical School, University of Texas Health, Houston, TX USA; 5 School of Dentistry, University of Texas Health, Houston, TX USA; 6 University of Wisconsin School of Medicine and Public Health, Madison, WI USA; 7 Present Address: Children’s Wisconsin and Medical College of Wisconsin, Milwaukee, WI USA

**Keywords:** Achondroplasia, Anthropometry, Height/length, Weight, Head circumference

## Abstract

**Background:**

Achondroplasia is the most common genetic skeletal disorder causing disproportionate short stature/dwarfism. Common additional features include spinal stenosis, midface retrusion, macrocephaly and a generalized spondylometaphyseal dysplasia which manifest as spinal cord compression, sleep disordered breathing, delayed motor skill acquisition and genu varus with musculoskeletal pain. To better understand the interactions and health outcomes of these potential complications, we embarked on a multi-center, natural history study entitled CLARITY (achondroplasia natural history study). One of the CLARITY objectives was to develop growth curves (length/height, weight, head circumference, weight-for-height) and corresponding reference tables of mean and standard deviations at 1 month increments from birth through 18 years for clinical use and research for achondroplasia patients.

**Methods:**

All available retrospective anthropometry data including length/height, weight and head circumference from achondroplasia patients were collected at 4 US skeletal dysplasia centers (Johns Hopkins University, AI DuPont Hospital for Children, McGovern Medical School University of Texas Health, University of Wisconsin School of Medicine and Public Health). Weight-for-age values beyond 3 SD above the mean were excluded from the weight-for-height and weight-for-age curves to create a stricter tool for weight assessment in this population.

**Results:**

Over 37,000 length/height, weight and head circumference measures from 1374 patients with achondroplasia from birth through 75 years of age were compiled in a REDCap database. Stature and weight data from birth through 18 years of age and head circumference from birth through 5 years of age were utilized to construct new length/height-for-age, weight-for-age, head circumference-for-age and weight-for-height curves.

**Conclusion:**

Achondroplasia-specific growth curves are essential for clinical care of growing infants and children with this condition. In an effort to provide prescriptive, rather than purely descriptive, references for weight in this population, extreme weight values were omitted from the weight-for-age and weight-for-height curves. This well-phenotyped cohort may be studied with other global achondroplasia populations (e.g. Europe, Argentina, Australia, Japan) to gain further insight into environmental or ethnic influences on growth.

**Supplementary Information:**

The online version contains supplementary material available at 10.1186/s13023-021-02141-4.

## Background

Monitoring growth of populations with short stature skeletal dysplasias presents challenges, given that the condition is relatively rare and relevant normative growth curves had, until recently, been limited to hand-smoothed curves derived from US data published in 1977 and 1978 [1, 2] Subsequent growth references have been produced from populations with achondroplasia in the US [3–7], Europe [8, 9], Australia [10, 11], Argentina [12–14], Egypt [15] and Japan [16]. These curves have been derived from a mix of longitudinal, cross-sectional, retrospective and/or prospectively collected data from populations ranging in size from 23 to 466 subjects, collected as long ago as 1967 and as recently as 2019.

Growth curves published by Horton et al. [2] included height, growth velocity, and head circumference and utilized mixed cross-sectional and longitudinal data from 400 individuals to construct hand-smoothed isopleths for the mean, ± 1, and ± 2 SD by age and sex. The next publication by Tachibana et al. [16] included only height data derived from a national survey in Japan. These data were the reference against which subsequent Japanese achondroplasia populations treated with growth hormone and/or limb lengthening were compared [17, 18]. More recently, Hoover-Fong et al. published weight-for-age [5], body mass index (BMI) [6] and height-for-age curves [4] from data extracted from a single clinical site and assessed by a single observer within the US, using a non-parametric smoothing approach. del Pino et al. published extensively on a population with achondroplasia in Argentina, including growth curves for weight, height, head circumference [14] and height velocity [12, 13] using the LMS approach. Tofts et al. have published similar growth charts, with the addition of BMI, in Australia, also using the LMS approach [10]. Ismail et al. utilized cross-sectional anthropometry from children 1–8 years of age to create growth curves for length/height, weight and head circumference in Egypt. The mean and standard deviation for each parameter at each year of life by sex was calculated and graphically smoothed with a polynomial fitting equation [15]. Finally, Merker et al. [8] derived curves for similar outcomes in a northern European population using a generalized additive model for location, scale, and shape (GAMLSS) approach. In addition to height, weight, head circumference and BMI, they also presented new curves for sitting height, leg length, arm span and foot length [9].

We have since amassed what is likely to be the largest dataset of anthropometric measures in patients with achondroplasia as one of the primary study domains of the multi-center study entitled CLARITY (Achondroplasia Natural History Study) [19]. Across four study sites we accrued length/height, weight and head circumference measures from 1374 subjects, totaling over 37,000 data points. Anthropometry data were consolidated from all available patient records which provided linked medical history (e.g. gestational age), surgical history (e.g. limb lengthening, cervicomedullary decompression) and medical treatments (e.g. growth hormone therapy). These ancillary data will ultimately allow us to consider the association of these medical conditions and treatments with respect to anthropometric status relative to these novel curves. Another unique opportunity this large cohort provides is to examine secular trends in weight in the US general population since there has been a clear trend of increasing body weight over the last several decades [20, 21].

The main objective of this study is to build on previous endeavors and derive updated growth references for length/height and weight from a US population, with corresponding tools for calculating sex- and age-specific Z-scores. Additionally, we provide new weight-for-length/height and head circumference growth references for US patients with achondroplasia. These parameters are compared to contemporary growth curves from global achondroplasia populations.

## Results

**Fig. 1:**
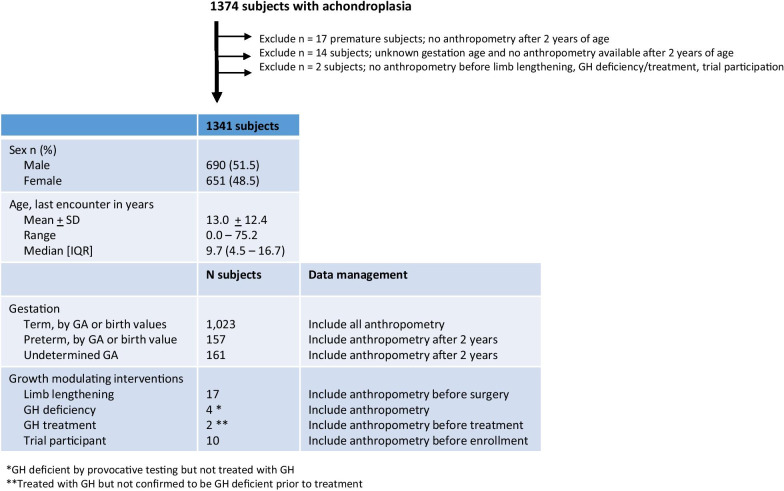
Description of subjects and criteria to include anthropometry in height/length-, weight- and head circumference-for-age and weight-for-age curves

There are 1374 total subjects with achondroplasia enrolled in this natural history study. Thirty-three subjects were excluded from further consideration in this analysis due to a lack of anthropometry data in various circumstances outlined in Fig. 1. Therefore 37,349 anthropometric data points of length/height, weight and head circumference from 1341 subjects (99.3% of the cohort) were available for construction of the curves presented here. Fifty-one percent of the subjects were male and all participants were an average age of 13.0 years + 12.4 [median 9.7; range 0–75.2] at their last encounter. The demographic background of this population is as follows: white (78.4%), Hispanic/Latinx (7.8%), black (6.1%), Asian (5.8%), Native American (0.2%), Hawaiian (0.07%) and unknown (1.6%) with some subjects represented in more than one category. All available anthropometric measures are included from individuals with reported term gestation (1024 subjects). Anthropometry from 157 premature subjects (i.e. gestational age < 37 weeks) were included after 2 years of age. For the 161 subjects with unknown gestational age, none had birth parameters available to assess against normal term infant values. Therefore, anthropometry measures were included only after 2 years of age for the 161 subjects with unknown gestational age. Of those who underwent growth modulating surgery or treatment, anthropometry were included from before these activities, but not after.

The total number of subjects and the number and type of anthropometric data points contributed by age group are shown in Table 1.

**Table 1:** Number of subjects and length/height, weight and head circumference data points contributed by sex at non-mutually exclusive age intervals corresponding to curves

	Birth		Birth to < 3 years				2–18 years				Birth to < 5 years				> 18 Years			
	Subjects (n) points (n)		Subjects (n)		Points (n)		Subjects (n)		Points (n)		Subjects (n)		Points (n)		Subjects (n)		Points (n)	
	M	F	M	F	M	F	M	F	M	F	M	F	M	F	M	F	M	F
Length/height	400	361	544	489	3024	2700	549	503	4011	3709	574	523	4102	3626	129	149	297	355
Weight	471	421	550	493	3602	3107	531	484	4553	4263	570	521	4830	4177	129	148	475	519
Head circumference	232	189	498	441	2643	2331					534	489	3449	3012				

Time points and age intervals include birth, birth to < 3 years of age, 2-18 years, birth to < 5 years and > 18 years. The number of subjects and data points (i.e. length/height, weight and head circumference) represented in the corresponding growth curves are presented in this table by sex at non-mutually exclusive age intervals. Overlapping age intervals include birth, birth to < 3 years of age, 2–18 years, and birth to < 5 years

Table 2 illustrates the longitudinal nature of these data. Over half of this achondroplasia cohort contributed five or more data points in all three growth categories while approximately one-third of the population contributed ten or more data points in over each parameter.

**Table 2:** Longitudinal characteristics of the anthropometry data for 1,341 subjects with achondroplasia

	Length/height N (% of total population)	Weight N (% of total population)	Head circumference N (% of total population)
> 3 points	977 (71.1)	968 (70.5)	879 (64.0)
> 5 points	799 (58.2)	823 (60.0)	699 (50.9)
≥ 10 points	510 (37.1)	544 (39.6)	385 (28.0)

The majority of the anthropometry data were longitudinal in nature with over 500 subjects contributing 10 or more length/height and weight values and nearly 400 with 10 or more head circumference values

**Fig. 2:**
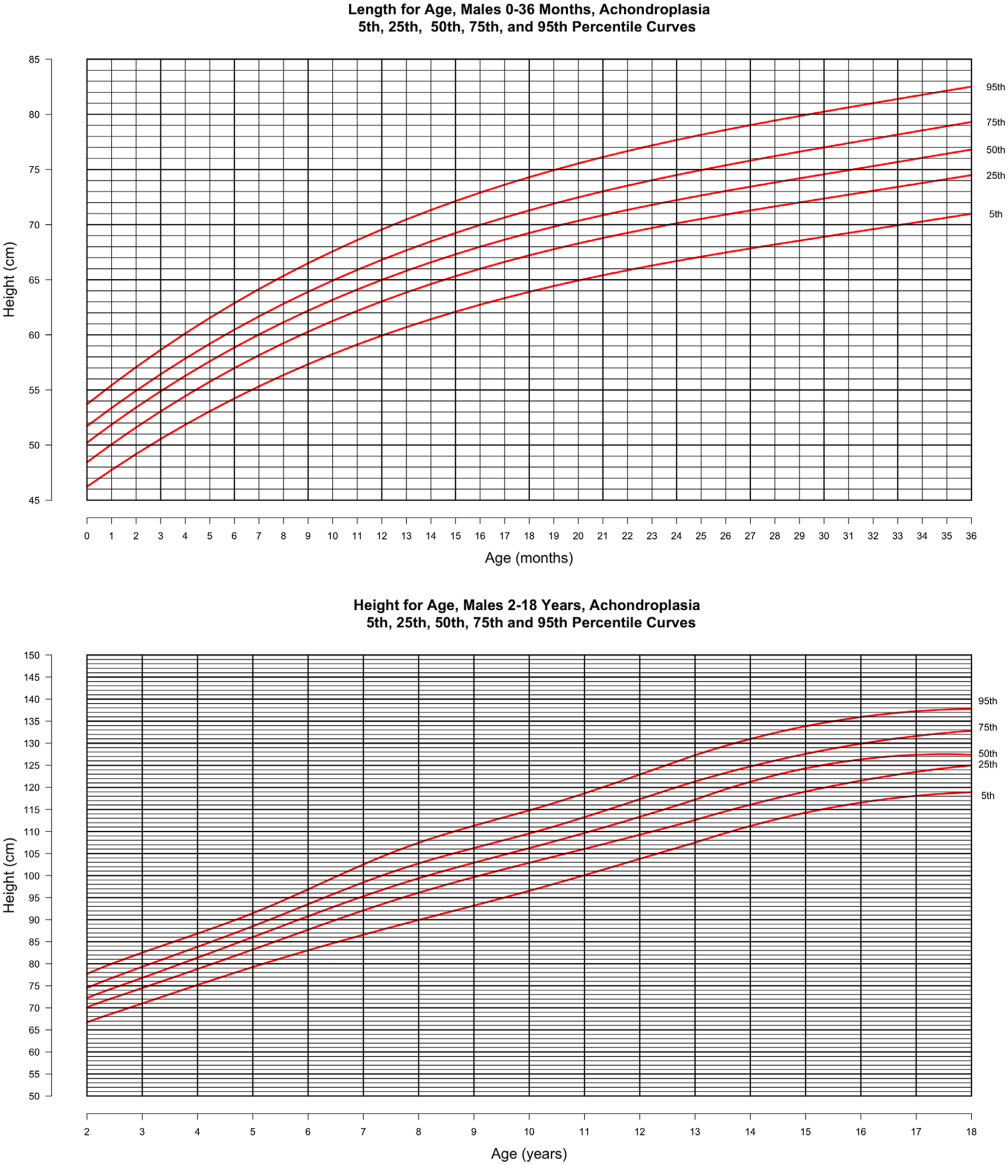
Stature-for-age for males with achondroplasia, 5th, 25th, 50th, 75th and 95th percentiles. Birth—36 months (top) derived from 3024 data points from 544 subjects and birth—18 years (bottom) derived from 4011 data points from 549 subjects

**Fig. 3:**
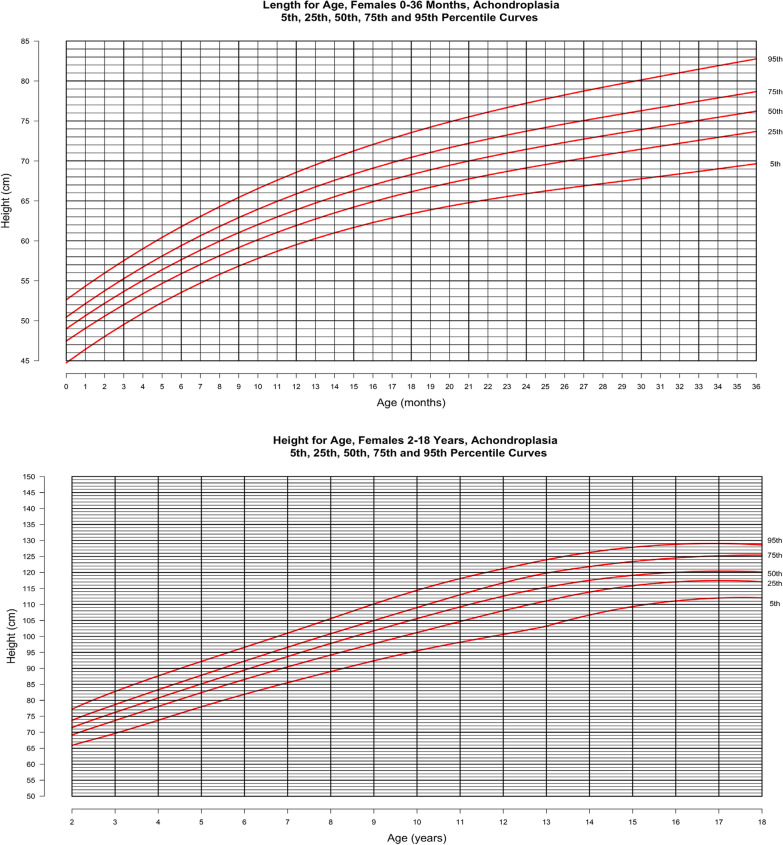
Stature-for-age for females with achondroplasia, 5th, 25th, 50th, 75th and 95th percentiles. Birth—36 months (top) derived from 2700 data points from 489 subjects and birth—18 years (bottom) derived from 3703 data points from 502 subjects

In Figs. 2 and 3, length for age is shown at the top (0–36 months) and height for age at the bottom (2–18 years) for males and females, respectively. In both figures, the curves represent the 5th, 25th, 50th 75th and 95th percentiles for the achondroplasia cohort. Final adult height is available from 277 individuals (128 males, 149 females) in this cohort, indicating average (SD) adult male height is 129.9 (6.25) cm [4′3.1″] and that for females is 122.4 (5.9) cm [4′0.2″].

**Fig. 4:**
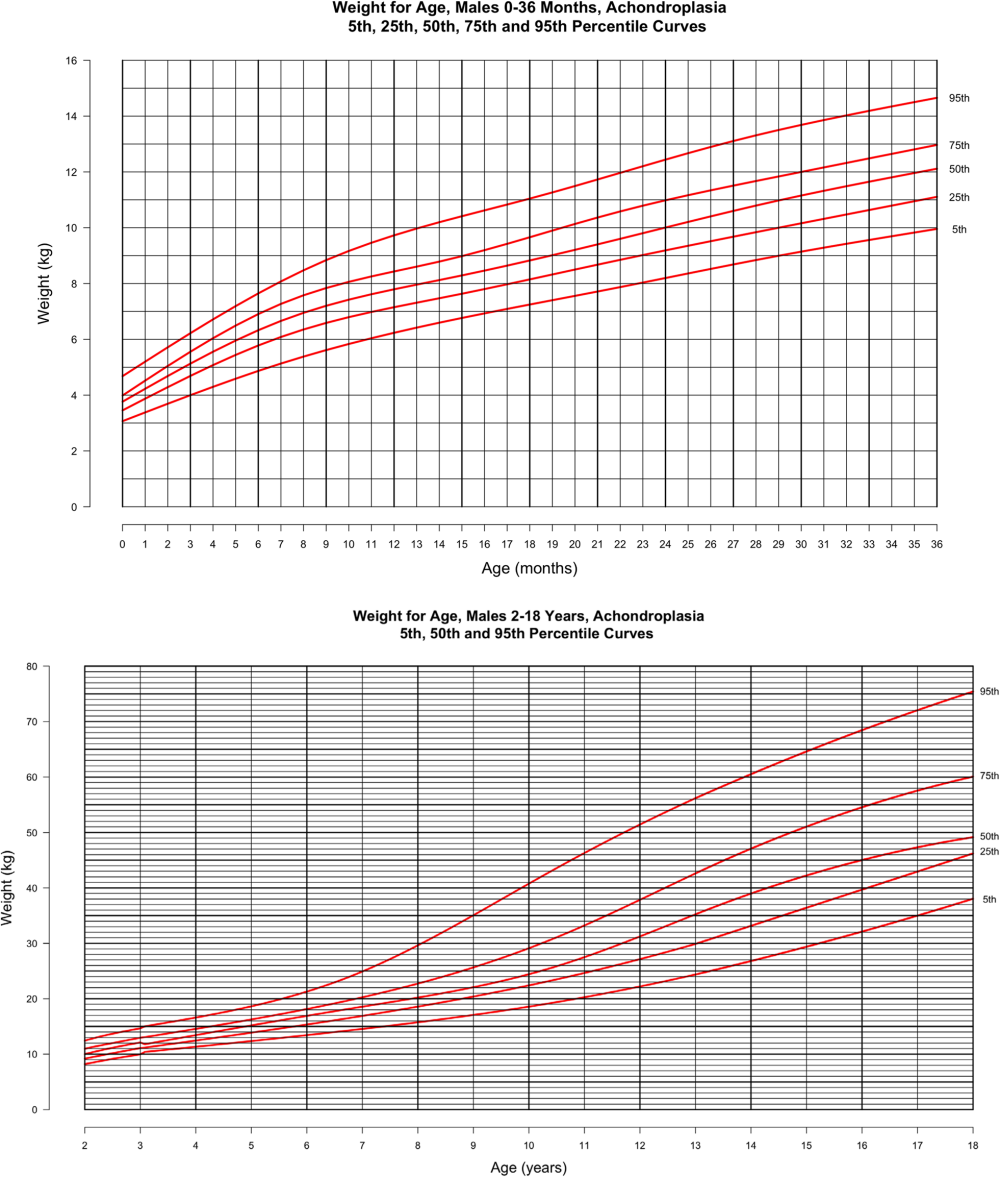
Weight-for-age for males with achondroplasia, 5th, 25th, 50th, 75th and 95th percentiles. Birth—36 months (top) derived from 3585 data points from 549 subjects and birth—18 years (bottom) derived from 4512 data points from 528 subjects

**Fig. 5:**
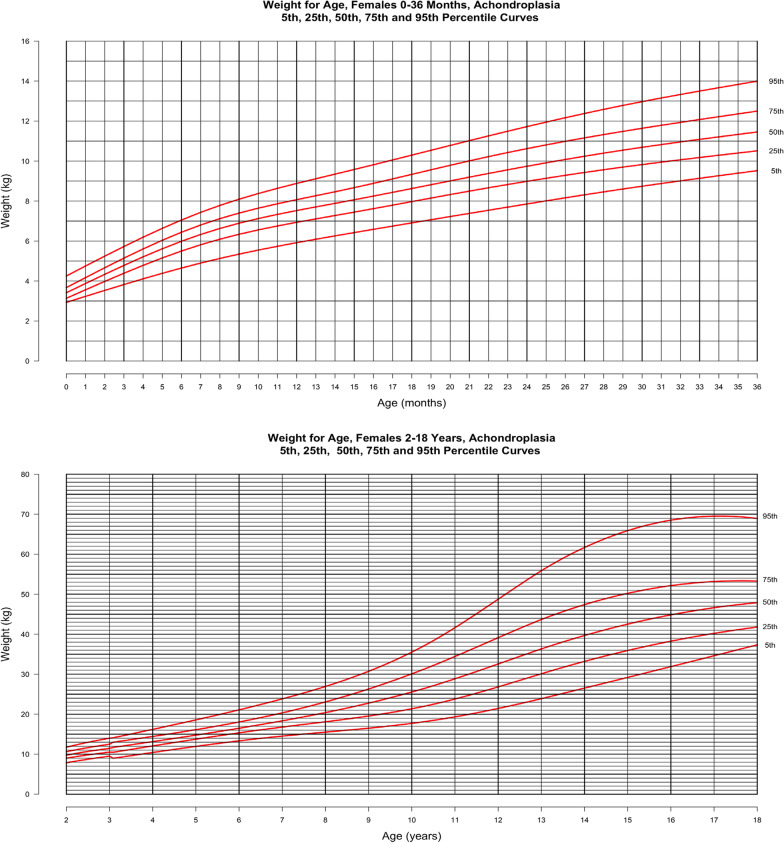
Weight-for-age for females with achondroplasia, 5th, 25th, 50th, 75th and 95th percentiles. Birth—36 months (top) derived from 3087 data points from 491 subjects and birth—18 years (bottom) derived from 4236 data points from 482 subjects

In Figs. 4 and 5, weight-for-age is shown at the top for the younger cohort and at the bottom for population from birth through 18 years. The curves represent the 5th, 25th, 50th, 75th and 95th percentiles derived from the raw weight data. For these curves, weight values > 3 SD above or below the mean were excluded. In males, this reduced the cohort from 550 subjects contributing 3602 weight data points to 549 subjects with 3585 data points in the birth to 3-year curves. In the curves for 2–18 year-old males, this exclusion decreased the total number of subjects and points contributed from 531 subjects with 4553 points to 528 subjects with 4512 points. In females from birth to 36 months, this exclusion decreased the group from 493 with 3107 weight for age points to 491 with 3087 points while in the older subcohort of 2–18 years there was a decrease in data points from 484 persons with 4263 points to 482 persons with 4236 data points. Utilizing the average weight values in the first year of life, the observed weight gain in males in this cohort is 21.2 g per day in the first month, 21.6 g per day in first to third months, 10.8 g per day in the third to sixth months, and 7.4 g per day in sixth to twelfth months. In females, the observed average weight gain is 21.2 g per day in the first month, 16.5 g per day in first to third months, 14.0 g per day in the third to sixth months, and 11.8 g per day in sixth to twelfth months.

Visualization of weight-for-age Z-scores by birth decade, 5-year age categories, and sex using boxplots indicated no evidence of consistent secular trends in weight in any age group for either males or females (Additional file 1: Fig. S1). However, subjects with extreme Z-scores (e.g. beyond − 3 or + 3 Z-score units) were apparent and were excluded from these weight-for-age curves and the subsequent weight-for-height curves.

**Fig. 6:**
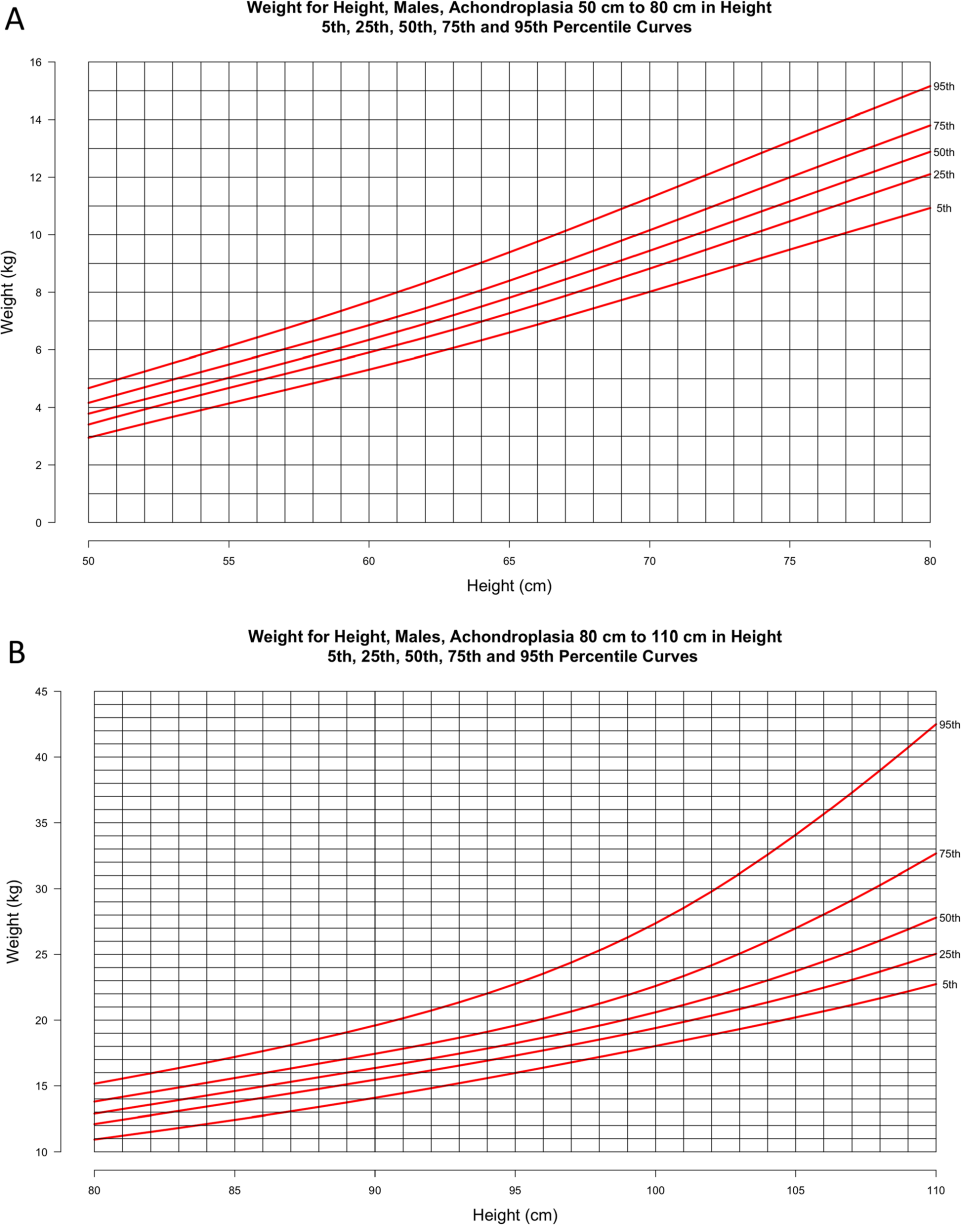

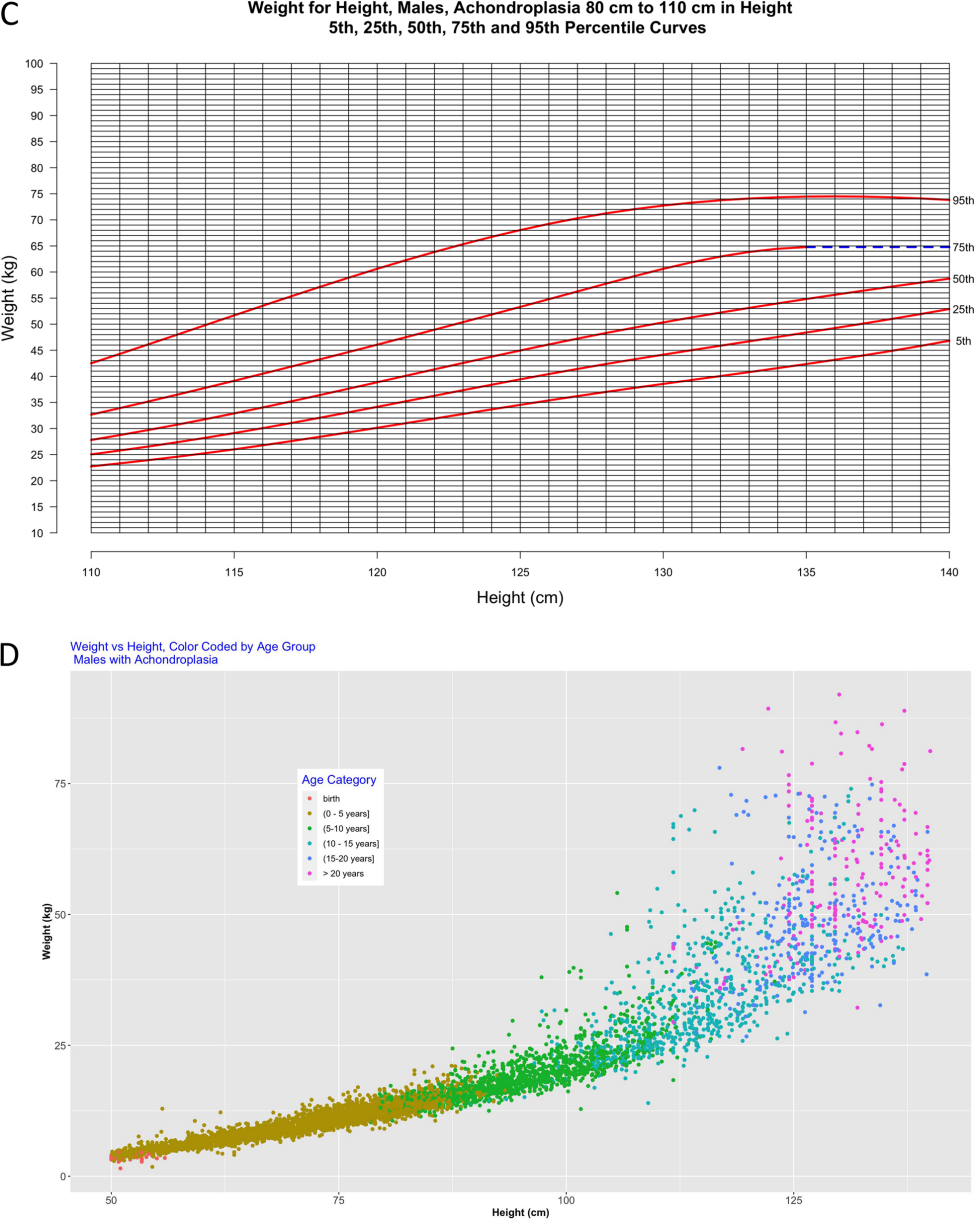
**a**–**d** Weight-for-height curves for males **a** ≥ 50 cm and < 80 cm including 2822 data points from 493 subjects and males **b** > 80 cm and < 110 cm including 2180 data points from 413 subjects; Weight-for-height curves for males **c** > 110 cm and < 140 cm including 956 data points from 265 subjects and **d** by age category

**Fig. 7:**
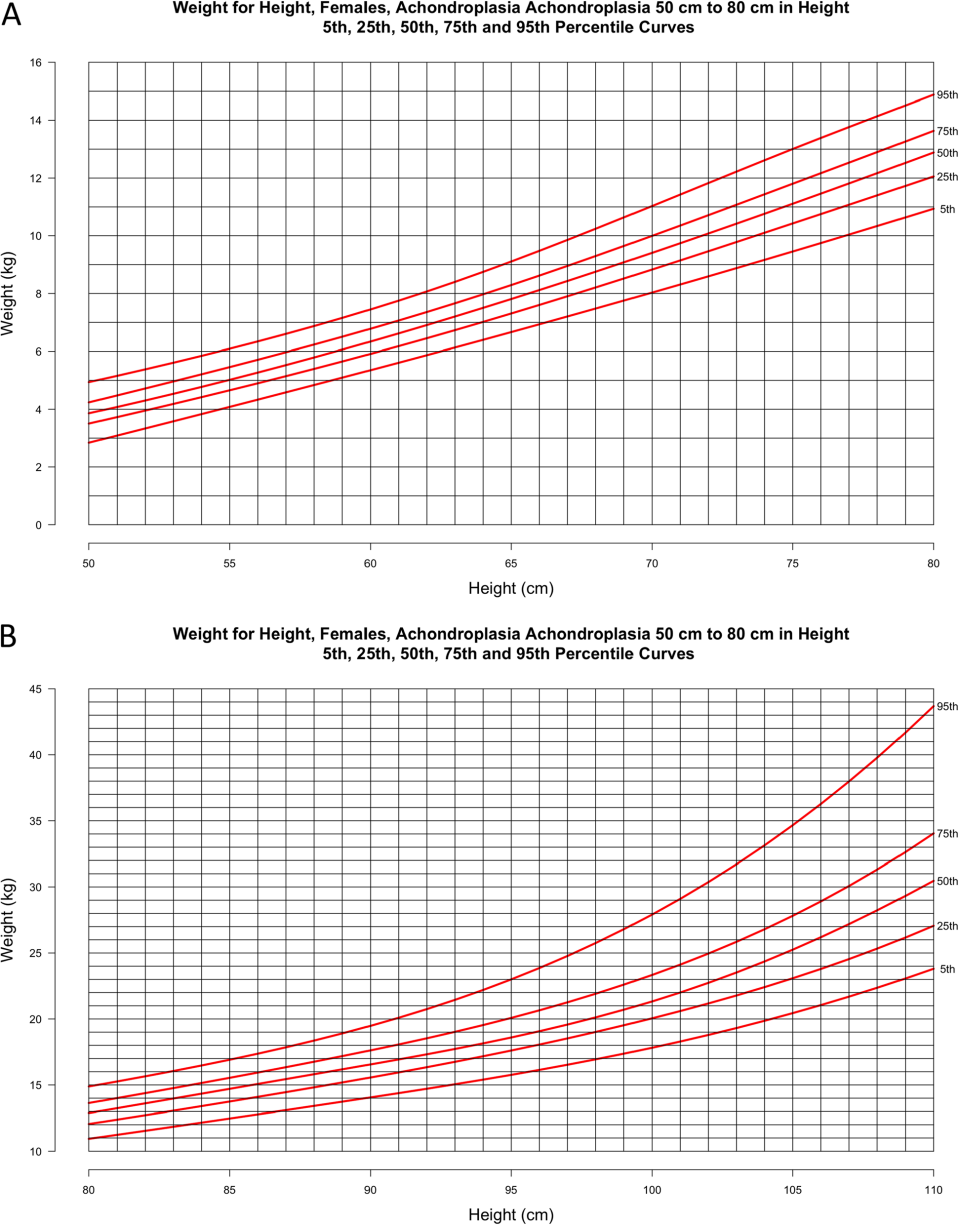

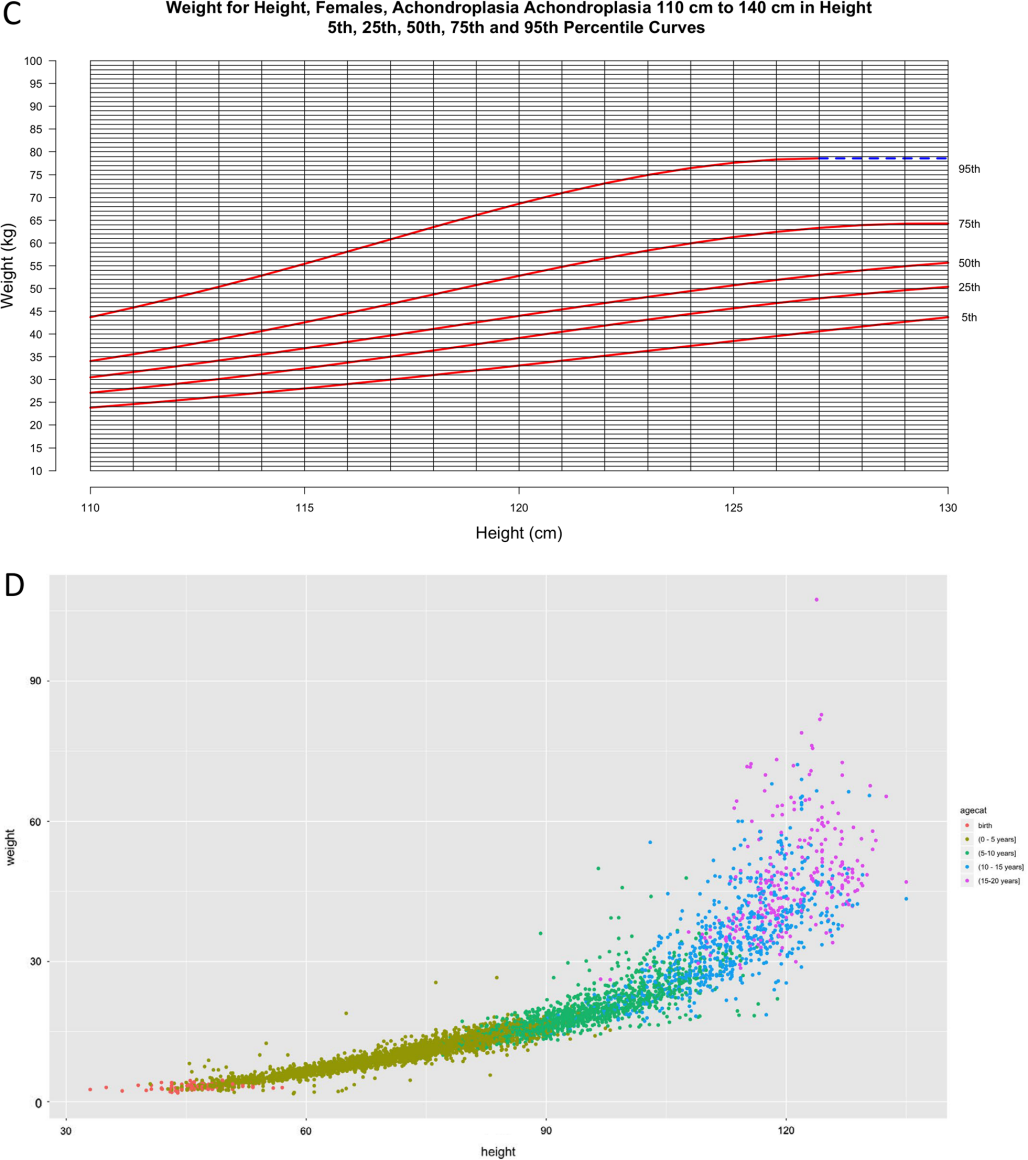
**a**–**d** Weight-for-height curves for females **a** ≥ 50 cm and ≤ 80 cm including 2478 data points from 440 subjects and females **b** > 80 cm and ≤ 110 cm including 2420 data points from 388 subjects.; Weight-for-height curves for females **c** ≥ 110 cm and ≤ 140 cm including 952 data points from 263 subjects and **d** by age category

Figures 6 and 7 present novel weight-for-height curves for males and females, respectively. These are divided into 3 groups based on height to allow for optimal resolution for clinical use.

**Fig. 8:**
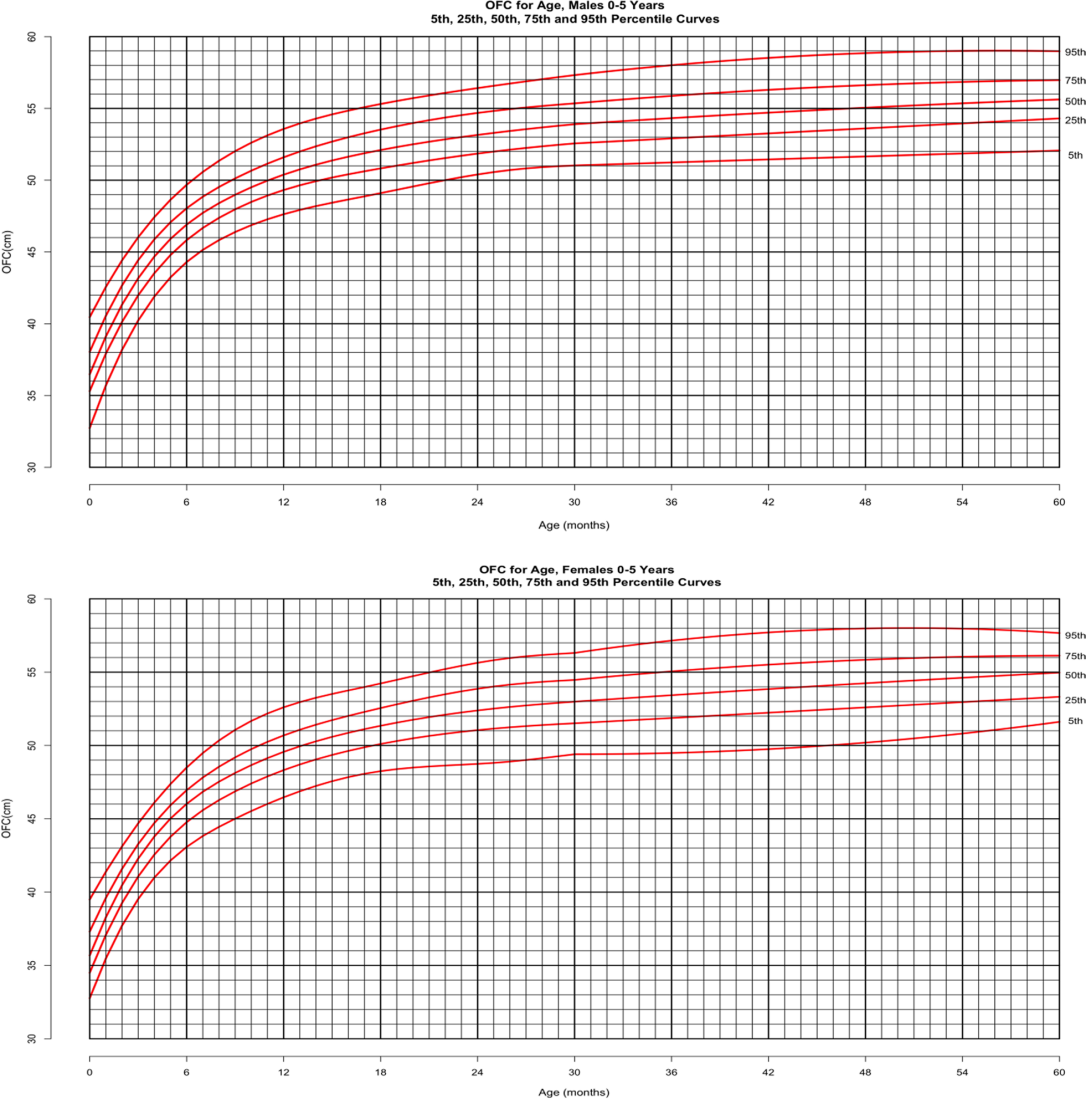
Head circumference-for-age from birth through 5 years for children with achondroplasia, 5th, 25th, 50th, 75th and 95th percentiles. Male curve (top) derived from 3449 data points from 534 subjects and female curve (bottom) derived from 3012 data points from 489 subjects. These figures include data points from subjects who underwent shunt placement and cervicomedullary decompression (CMD) (from before and after the procedures). There was no difference in the male or female head circumference-for-age curves when the data points from these subjects were excluded. For males, there were 361 data points from 49 subjects who had a shunt (196 data points after the surgery) and 825 data points from 116 subjects who had CMD (462 data points after the surgery). For females, there were 197 data points from 99 subjects (116 data points after the surgery) and 757 data points from 122 subjects who had CMD (374 data points after the surgery). This figures include data points from subjects who underwent shunt placement and CMD (from before and after the procedures). There was no difference in the male or female head circumference-for-age curve when the data points from these subjects were excluded. For males, there were 361 data points from 49 subjects who had a shunt (196 data points after the surgery) and 825 data points from 116 subjects who had CMD (462 data points after the surgery). For females, there were 197 data points from 99 subjects (116 data points after the surgery) and 757 data points from 122 subjects who had CMD (374 data points after the surgery)

Figure 8 shows the head circumference data from birth through 5 years of age for males (top) and females (bottom) including the 5th, 25th, 50th, 75th and 95th percentiles. Head circumference data of premature infants (i.e. born ≤ 37 weeks gestation) were deleted from curves from birth through 2 year of age. Otherwise, all data points were included in the head circumference curves presented here regardless of surgical history involving the foramen magnum, upper cervical spine and ventricular shunting. Sensitivity analysis of the curves created by maintaining the anthropometry from all subjects undergoing these surgical procedures versus the curves derived after omitting their data indicates there is no difference between the curves. In this achondroplasia cohort, there is minimal head circumference growth after 3 years of age. For males, 90% of the median head circumference for 5-year-old males is achieved at 13 months of age and that for females is achieved at 12 months of age.

Additional file 2: Table S1 includes the mean and SD values for Z-score calculations for males and females, respectively, for length/height, weight and head circumference from birth through 18 years. Additional file 3: Table S2 includes the mean and SD values for Z-score calculations for males and females for weight-for-length. Note that the male height values in this table range from 50 to 140 cm while that for females are from 50 to 134 cm due to a paucity of data beyond this upper limit of height.

## Discussion

Growth reference data presented here add to an expanding literature in this area, with the potential to benefit patient care and enhance research into qualities of growth among infants, children, and teens with achondroplasia. The large number of data points amassed enhance growth curves that our research group previously established based on a single data source [4–6]. We also explored the possibility of secular trends in weight in children by age group and decade of birth and utilized weight-for-age data to screen for extremes in weight that were excluded from our novel, prescriptive weight-for-age and weight-for-length/height curves. We expect these figures to have clinical utility for identifying children carrying excess weight for their stature. Additionally, we add to the literature by offering new head circumference curves for young children from the US. We expect the utility of this large achondroplasia anthropometric database in clinical care and research to be multi-fold.

As supported by the comparison of height trajectories to that of average stature children, the achondroplasia-specific *FGFR3* mutation influence on linear growth is, of course, greater than any environmental or ethnic factor that could be derived from other studies of linear growth in achondroplasia. By comparing to other studies of linear growth in achondroplasia (e.g. from Europe, Argentina, Australia, Japan) [8, 10, 14, 16], more subtle environmental or ethnic influences on attained height for a given age across childhood could potentially be ascertained. Based on visual inspection of the most recently published height-for-age curves, median height was similar across populations at 5 and 10 years of age, ranging from ~ 85 to 87 cm in girls and 86–88 cm in boys at 5 years and 104–106 cm in girls and 106–108 cm in boys at 10 years. At 15 years, median height in Argentinian girls was at least 3 cm shorter than peers from the US, Australia, and Europe (116 vs. 119–121 cm), as previously noted [14]. However, at 18 years, the oldest age for which data were available for all settings, both the US and Argentinian girls, at a median height of ~ 120 cm, were shorter than their European peers (124 cm). For boys, median heights were quite similar across settings at 15 years (123–125 cm), but variable at 18 years (127–134 cm; shortest in the US, tallest in Australia). Additional file 4: Table S3 summarizes the length/height and weight data from the achondroplasia populations presented in this manuscript as well as those from Europe [8], Australia [10] and Argentina [14].

Greater variability in the older age groups could reflect underlying genetic differences in growth potential, environmental influences on growth, different timing of pubertal development (although any pubertal gains in height seem subtle), a relative paucity of data and selection bias at older ages, or different modelling approaches that contribute to variability in estimates of median height. All of these possibilities need to be studied in a more rigorous manner. For average stature children, a global growth standard is used to characterize length/height in children 0–5 years [22], in whom growth occurs quite comparably regardless of ethnic background when unrestricted by environmental constraints [23]. A reference based largely on US-derived data is also used globally to characterize heights of average stature children 5–20 years of age [24], although the assumption of comparability of growth trajectories through childhood and adolescence by background is not certain [25]. Ultimately, we may find that compiling height-for-age across populations could be used to generate a global standard for achondroplasia.

Additionally, whether puberty results in a linear growth spurt, and whether its timing, tempo, or magnitude varies by ethnicity or other environmental factors remains an area requiring resolution. The cross-sectional presentation of height-for-age data does not suggest a substantial pubertal growth spurt in achondroplasia, although others have speculated that one exists based on the application of height data collected serially in a small number of adolescents with achondroplasia to the Preece–Baines model [13]. That conclusion may be an artifact of the modeling approach, and if a pubertal spurt in linear growth does occur it is very modest. A harmonized effort across populations to collect longitudinal Tanner stage data in conjunction with height velocity would elucidate effects of puberty on height gains in achondroplasia, but this question is not resolvable in this analysis of retrospective growth data.

Accumulation of excess weight is a concern in the population with achondroplasia [5, 6]. The growth curves presented here, as shown previously [6], show more overlap in weight than height with the average stature population-findings consistent with a body morphology with a high ratio of trunk to extremity size. Additionally, however, excess adiposity is a concern in this population, with few tools available to guide what optimal weights should be. Given the known obesity epidemic in the US it is useful to compare weight-for-age across populations to ascertain whether the US population with achondroplasia is heavier than their global counterparts. In fact, also based on visual inspection of most recent growth curves [8, 10, 13, 14], median weights were similar across settings until 10 years of age in girls (14–16 kg at 5 years; 24–26 kg at 10 years) and boys (15–16 kg at 5 years; 24–26 kg at 10 years), while US girls from this study were heavier at 15 years (43 kg vs. 36–40 kg) and 17 years (last age in common across settings; 47 kg in US vs. 41–44 kg from other sites). Among boys, US and European boys were comparable at 15 years (42 kg vs. 34 kg in Australia and 37 kg in Argentina) and 17 years (47 kg in the US, 48 kg in Europe vs. 38 kg in Australia and 42 kg in Argentina). Although findings are subject to the same caveats described above that could account for variability in height-for-age across settings, they are also consistent with children with achondroplasia being heavier-set in the US, particularly for their stature, than elsewhere, with the possible exception of Europe. This is despite our efforts to screen out extremes in weight. We were surprised that secular trends in excess weight by decade of birth in our population were not found, but did note a large number of extreme weights for a given height. In order to avoid skewing reference data to higher than optimal weights, potentially giving patients the impression that a high weight is acceptable, we utilized the weight-for-age curves generated here to remove cases with extreme weights before generating novel weight-for-stature charts that we propose for clinical use in this population.

We propose new weight-for-stature charts to help clinicians integrate information on weight and height of patients to improve guidance for achieving appropriate weights in a patient population prone to obesity. We [6] and others [8, 10] had also established BMI-for-age curves, which integrate information on weight and height by age. However, BMI can be difficult to interpret, and it can be tempting to use inappropriate cutoffs that have been perpetuated in the literature for average stature individuals to misattribute overweight and obesity to patients with achondroplasia [26]. Additional studies are needed to quantify body composition in individuals with achondroplasia and correlate these values with BMI.

Sensitivity analysis of the head circumference data indicated there was no difference in the curves generated from all available data and those derived from data excluding those undergoing foramen magnum/C1/C2 decompression and/or ventricular shunting. Therefore, we opted to include all available head circumference data to derive these new curves. These curves may now be utilized in the clinic and research venues to ascertain deviation from normal cranial growth which should prompt further investigation.

Limitations of the study include the fact that data were not collected at pre-determined time intervals, due to the clinic-based and retrospective nature of the study. Regrettably, we do not have sufficient Tanner staging available in this cohort to compare landmarks of pubertal development against growth. Clearly this should be ascertained in future cohort studies.

Strengths of this study include the fact that in this large, well-phenotyped cohort of patients with achondroplasia, we could eliminate the potential confounding effect of surgical and medical interventions influencing linear growth by omitting the stature data collected after these procedures. In this regard, the curves represent a norm against which individuals and populations who undergo novel medical therapies and surgical interventions to ascertain effects of those treatments can be compared. Although these data were collected by multiple providers and therefore potentially subject to inconsistencies, these growth parameters were all collected in skeletal dysplasia clinics or affiliated clinical site where care providers are accustomed to performing anthropometry. Additionally, the large overall amount of anthropometric data and the fact that over 50% of the cohort contributed 5 or more data points for all 3 parameters means it is more longitudinal than cross-sectional in nature and therefore more reflective of longitudinal growth. Finally, the methodology employed to generate our curves by modeling separate splines over the age range accounted for the fewer data points available at older ages.

To the last point, we offer a note regarding the choice to use penalized cubic p-splines to estimate age (and height with the weight for height analyses) percentiles, means and standard deviation as compared to LMS (Box–Cox) employed by others. Our approach requires no assumptions about the age specific anthropometric measurement distributions. The LMS method uses a normalizing power transformation of age-specific anthropometric measures which requires the estimation of three age-specific parameters: L (lambda), the transforming power, M (mu), the age specific median, and S (sigma) the age specific standard deviation. The resulting percentile estimates based on this approach are sensitive to these estimates. The LMS approach is appropriate when there are a relatively large number of measurements in each 1-month age interval, allowing for estimates of these 3 parameters at each age. However, as noted in our data, the number of measures per age-interval is variable and decreases substantially with increasing age. Thus, our smoothing approach was appropriate given the characteristics of available data.

## Conclusion

In summary, the utility of this large achondroplasia anthropometric database permeates clinical care and research endeavors for our patients. Exclusion of weight-for-age values beyond 3 + SD in these novel weight-for-stature and weigh-for-age curves provide a stricter tool for weight assessment in this population. Mean and SD values of weight, stature and head circumference in a tabular form at 1 month intervals from birth through 18 years are offered for the first time for clinical and research applications. This large US achondroplasia population also may be studied in relation to other global achondroplasia populations (e.g. Europe, Argentina, Australia, Japan) to gain further insights into environmental or ethnic influences on growth. This large, well-phenotyped cohort of patients with achondroplasia may also be contrasted to individuals and populations who undergo novel medical therapies and surgical interventions to ascertain the effect of those treatments.

## Materials and methods

This study was approved by the Institutional Review Boards of Johns Hopkins University, AI DuPont Hospital for Children, McGovern Medical School University of Texas Health Science Center at Houston and University of Wisconsin School of Medicine and Public Health. The data collection methods have been described [19]. In brief, medical records of all available patients with achondroplasia (1957–2017) from the participating medical centers were abstracted for recorded anthropometric measures. A diagnosis of achondroplasia was made by molecular and/or clinical means by a clinical geneticist with expertise in the care of patients with skeletal dysplasias at the participating sites. Patients currently followed at any site signed a consent form for their data to be included in the database. For clinically inactive patients, a waiver of consent was used for inclusion. Data were populated in a REDCap database by study coordinators at each site [27], then downloaded as needed for analysis in R [28–30]. Percentile curves for stature and weight with corresponding tools for calculating sex- and age-specific Z-scores are presented. Additionally, we provide new weight-for-length/height and head circumference-for-age curves for US patients.

In each clinic, length was obtained in a supine position until at least 2 years of age or until the patient could stand for height measurements with a stadiometer. Weight was obtained with a standard clinical scale and head circumference was measured with a tape measure by a physician or healthcare provider at the dysplasia center or affiliated clinical site.

Exploratory analysis of the anthropometric data was performed within-person and by group over multiple parameters (e.g. by study site, sex, age category). Values violating predetermined criteria indicative of potential data error were verified against the primary medical records at each participating site. The data cleaning criteria necessitating further investigation included: stature difference > 5 cm between any 2 consecutive values; height > 139.9 cm, weight > 69 kg, weight difference > 5 kg between 2 values obtained in < 6 months, and head circumference > 60 cm or a difference between 2 consecutive points > 3 cm within 6 months. Additional algorithms to identify outlier data points were applied to flag potential erroneous values. Unverifiable or implausible values were removed. Anthropometry measurements acquired < 15 days apart were averaged and a single value was used for analysis.

Anthropometry from subjects born at term (≥ 37 weeks gestation) were included in the curves. Similar to the reference curves for average stature children in the US [31], anthropometry from subjects born prematurely were omitted from all curves up to 2 years of age. Thereafter, anthropometric measures were included in the growth curves since the effect of prematurity on size and growth diminishes beyond 2 years of age [32, 33]. For subjects with unknown gestational age, the plan was to assess birth parameters against sex-specific achondroplasia growth curves and include all anthropometry from those with birth values above − 2SD from the mean (i.e. a surrogate indicator of term gestation). Anthropometry from subjects with unknown gestation and no birth parameters were included after 2 years of age. Growth data from subjects who underwent surgical limb lengthening, were treated with growth hormone or participated in clinical trials of investigational pharmaceuticals were also omitted from these anthropometric analyses after the point of treatment initiation. Sensitivity analyses were performed with inclusion and exclusion of these sub-cohorts in their respective curves.

Once weight-for-age data points were cleaned according to the aforementioned methods, these data were examined over the following age groups: birth weight, day 1–5 years, 5–10 years, 10–15 years, 15–20 years and over 20 years by birth cohorts (< 1980, 1980s, 1990s, 2000s, 2010s) for secular trends and outliers using boxplots. Weight-for-age data points more than 3SDs above and below the mean in this dataset were excluded from the weight-for-age curves and weight-for-age Z-score tables presented here. These conditions were intended to reduce the possibility that values for weight at a given age would be considered ‘appropriate’ despite being biased upward due to excess weight or downward due to unusually small size. Weight-for-height curves were computed using observations with height values between 50 and 140 cm in males and 50–130 cm in females, excluding weight values that were 3 SD above and below the mean for age.

Subjects were also coded by prior neurocranial surgical procedures, including cervicomedullary decompression and ventriculoperitoneal shunt placement. Sensitivity analyses on the head circumference curves with and without data points obtained after neurocranial surgery were performed.

### Analysis

Descriptive statistics of the total study cohort were considered in the construction of these curves including sex, age at last measurement, gestational age, prior limb lengthening, growth hormone deficiency/treatment, and/or trial participation. For continuous measures, means and standard deviations, medians and interquartile ranges, and/or ranges were determined, and for binary and categorical measures, proportions.

Sex-specific birth length, weight and head circumference mean, standard deviation and 95% confidence intervals were computed for length, weight and OFC. These resulting confidence intervals were compared to the same average-stature mean values from the WHO growth standards [34] as recommended for children up to 2 years of age [35, 36] to ascertain whether birth size was statistically significantly different than in average stature children. The CDC growth reference [31] was used as a comparator thereafter to ascertain differences in growth patterns between children with achondroplasia and the average stature population. Final adult height was also determined by sex for this cohort.

Age-specific mean weights were estimated separately by sex, using a penalized cubic-spline approach. Sex-specific separate upper and lower standard deviation values for weight were computed for values above and below the mean to address rightward skewness in weight measures. The “upper standard deviation” refers to age-specific standard deviations of weight values greater than their respective age-specific means (the “upper weights”). To estimate these values, the empirical standard deviations of these upper weight values were estimated at each month of age using all upper weight values within 2 months of the specified age, and these empirical values were smoothed as a function of age using a penalized cubic-spline approach. The “lower standard deviation” refers to age-specific standard deviations of weight values less than or equal to their respective age-specific means (the “lower weights”), and these were estimated via the same approach as the age-specific upper standard deviations. Age and sex specific Z-scores for all weight values were calculated based on empirical estimates of the weight-for-age means, and upper and lower standard deviations.

Secular trends in weight by birth decade, age and sex were visually inspected using boxplots of weight-for-age Z-scores. Z-scores were calculated using age- and sex-specific means and (upper or lower, depending on whether the individual weight value was larger or smaller that its age and sex-specific mean weight) standard deviations for weight from the aforementioned sample generated values. Extreme data points observed in all age groups (> 3 SD above the mean) were eliminated from the weight-for-age and weight-for-height curves. In this way, greater than average weight extremes were not allowed to divert the upper isopleths upward, thereby misrepresenting usual weights. Utilizing the average weight values in the first year of life, the expected/normal weight gain per day by sex in the first month, 3 months, 6 months and 12 months were also calculated.

Sex-specific percentile curves-for-age were generated for subjects from birth through 18 years for height and weight, and birth through 5 years for head circumference, via a multi-step process. First, age-specific empirical 5th, 25th, 50th, 75th and 95th percentiles for height and weight were estimated using values within an age-specific window-based approach at each month of age for the period of 0–18 years; age specific empirical percentiles for head circumference were estimated similarly, but only using data for the period of 0–5 years. For the first year of age, a window of ± 0.5 months was used to estimate the empirical month-specific percentile; for > 1–3 years, a window of ± 1 months was used; for > 3–18 years, a window of ± 3 months was used. More copious data at the younger ages allowed for narrower smoothing windows.

These empirical 5th, 25th, 50th, 75th and 95th percentiles were then modeled as a function of age. For the height-for-age curves, penalized cubic splines were used to estimate age-specific average height-percentile values across the entire 0 to 18-year domain. For head circumference, the same smoothing approach was taken using all data between 0 and 5 years to estimate the clinical head circumference-for-age curves. The weight-for-age data necessitated a stratified approach to smoothing because of the increasing skewness of weight values with increasing age. Separate penalized cubic smoothing splines were used to estimate the weight-for-age percentile curves for age intervals of birth through 3 years and 3–18 years. The number of knots were adjusted to balance model fit with enforced monotonicity of the weight-for-age percentile estimates. Finally, to maximize clinical utility, the predicted percentile values from the penalized cubic splines were fit to age via a two-degree polynomial for the height and weight results from 13 to 18 years, and for the head circumference results from 4 to 5 years. This approach was taken as a second round smoothing approach to average out the minor variations in the original predicted values over age.

Sex-specific weight-for-height curves were computed for heights between 50 and 140 cm for males and 50–130 cm for females. Empirical 5th,25th, 50th, 75th and 95th weight percentiles for each integer centimeter of height were estimated based on weight-measurements corresponding to height values within + /1 cm windows of each specific height index value. Penalized cubic splines were then used to estimate height-specific average weight-percentile values across each sex-specific height domain.

All analyses and graphs were done in R [29]. Reference tables of means and standard deviations derived from these stature, weight and head circumference curves at each month interval, and for weight-for-length/height, are included in the supplemental materials.

## Supplementary Information

**Additional file 1: Fig. S1.** Box plot of weight Z-score by sex by age cohort.

**Additional file 2: Table S1.** Male and female height, weight and head circumference mean and SD by month from birth through 20 years.

**Additional file 3: Table S2.** Male and female weight-for-height mean and SD by centimeter from 50–140 cm in males and 50–134 cm in females.

**Additional file 4: Table S3.** Comparison of anthropometry data from achondroplasia populations in this CLARITY study and from Argentina (del Pino), Australia (Tofts) and Europe (Merker).

## Data Availability

The datasets generated and/or analyzed during the current study are not publicly available but are available from the corresponding author on reasonable request.
