# Supplementary figures and images for: Growth in achondroplasia including stature, weight, weight-for-height and head circumference from CLARITY: achondroplasia natural history study—a multi-center retrospective cohort study of achondroplasia in the US
Source: Orphanet J Rare Dis. 2021 Dec 23;16:522. doi: 10.1186/s13023-021-02141-4 (PMC8697459; doi:10.1186/s13023-021-02141-4)

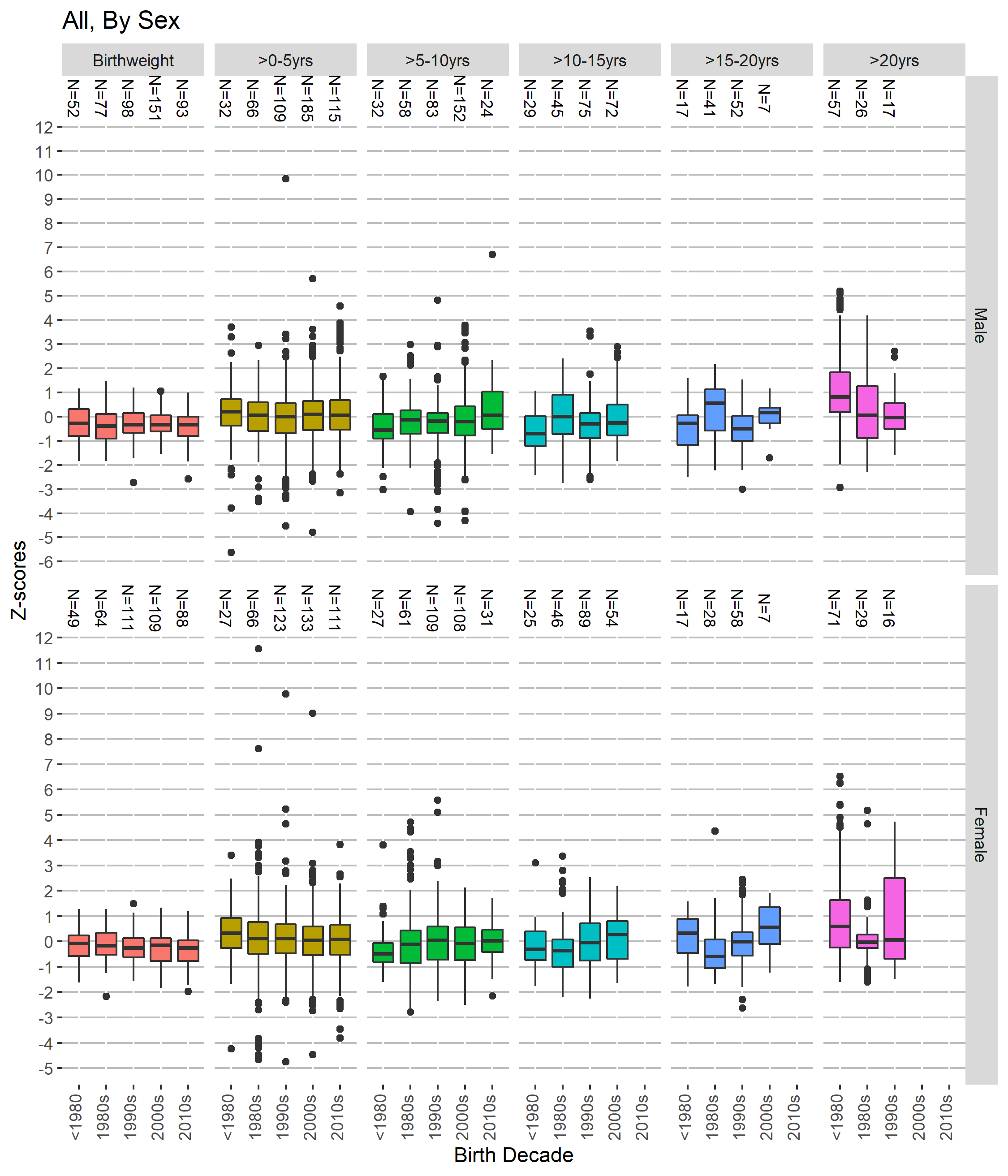

Supplement: Supplementary file 1 — Additional file 1: Fig. S1. Box plot of weight Z-score by sex by age cohort. [file 13023_2021_2141_MOESM1_ESM.tiff]
